# Supplementary material for: Visualization of Swift Ion Tracks in Suspended Local Diamondized Few-Layer Graphene
Source: Materials (Basel). 2023 Feb 7;16(4):1391. doi: 10.3390/ma16041391 (PMC9964662; doi:10.3390/ma16041391)
Supplement: Supplementary file 1 [file materials-16-01391-s001.zip › materials-2140010-supplementary.pdf]

Visualization of Swift Ion Tracks in Suspended Local Diamondized Few-Layer Graphene

Nadezhda A. Nebogatikova [1,*], Irina V. Antonova [1,2], Anton K. Gutakovskii [1,3], Dmitriy V. Smovzh [4], Vladimir A. Volodin [1,3] and Pavel B. Sorokin [5,*]

[1] Rzhanov Institute of Semiconductor Physics of the Siberian Branch of the RAS, Novosibirsk 630090, Russia; ifp@isp.nsc.ru (I.V.A.); inter@nstu.ru (A.K.G.); director@itp.nsc.ru (V.A.V.)
[2] Department of Semiconductor Devices and Microelectronics, Novosibirsk State Technical University, Novosibirsk 630087, Russia
[3] Physical Department, Novosibirsk State University, Novosibirsk 630090, Russia
[4] Kutateladze Institute of Thermophysics, Siberian Branch of the Russian Academy of Sciences, Novosibirsk 630090, Russia; rector@nsu.ru
[5] Technological Institute for Superhard and Novel Carbon Materials, Moscow 108840, Russia
* Correspondence: nadonebo@gmail.com (N.A.N.); pbsorokin@tisnum.ru (P.B.S.)

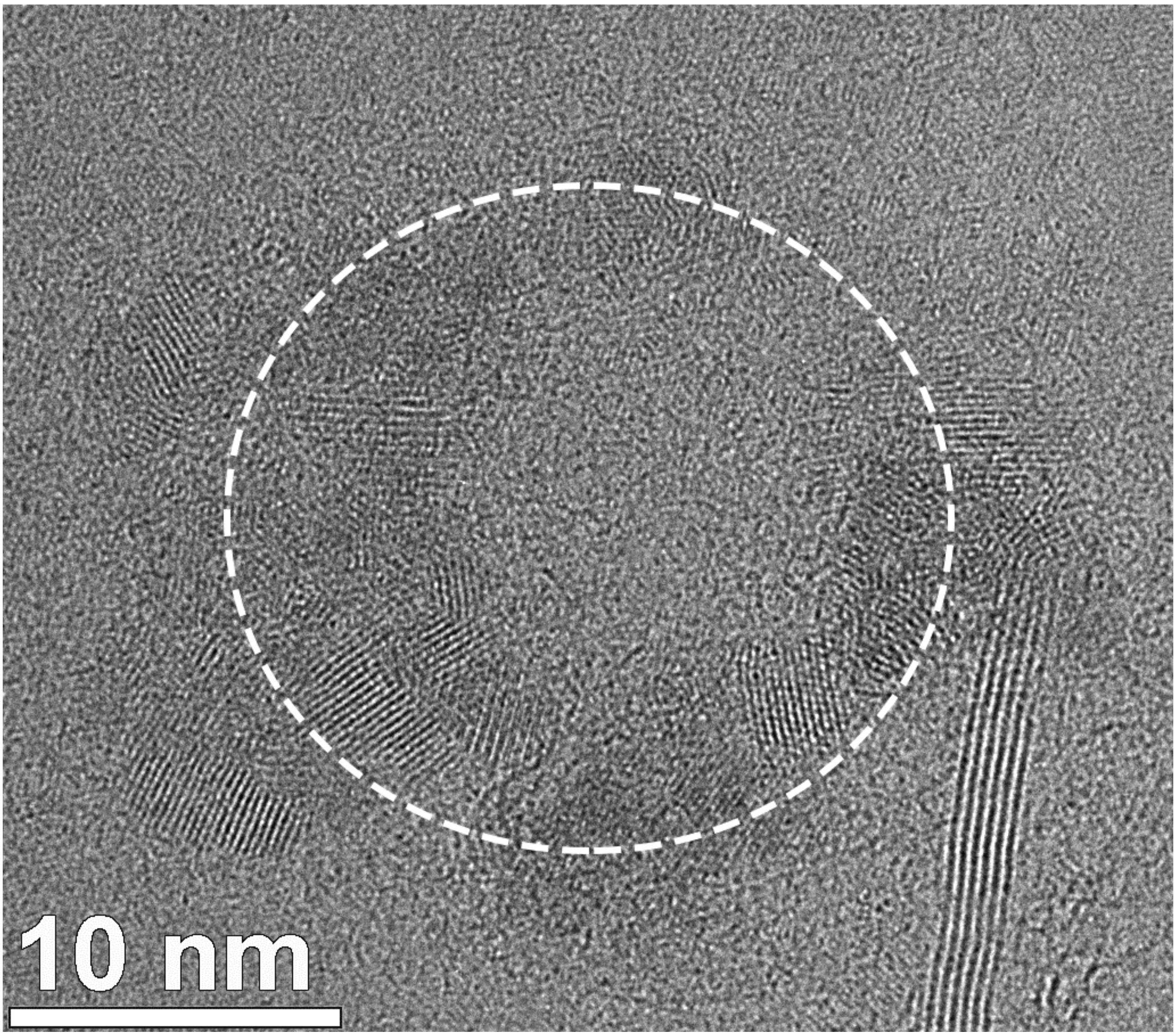

Figure S1: Cluster of nanocrystals at the periphery of the ion track at the irradiation dose of 1×10^{11} ion/cm^2, ion energy is 167 MeV.

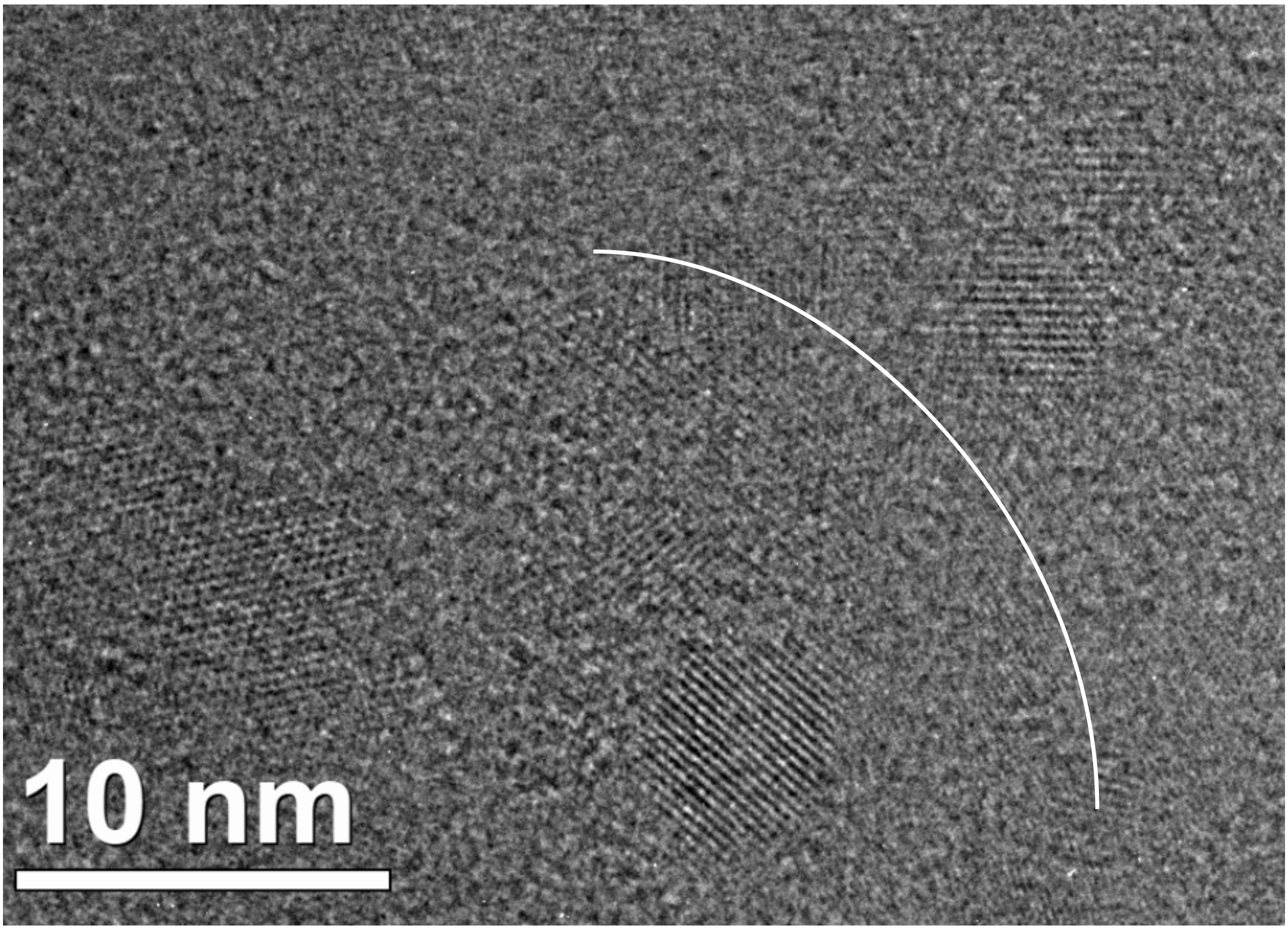

Figure S2: Cluster of nanocrystals at the periphery of the ion track at the irradiation dose of 1×10^{11} ion/cm^2, ion energy is 26 MeV.

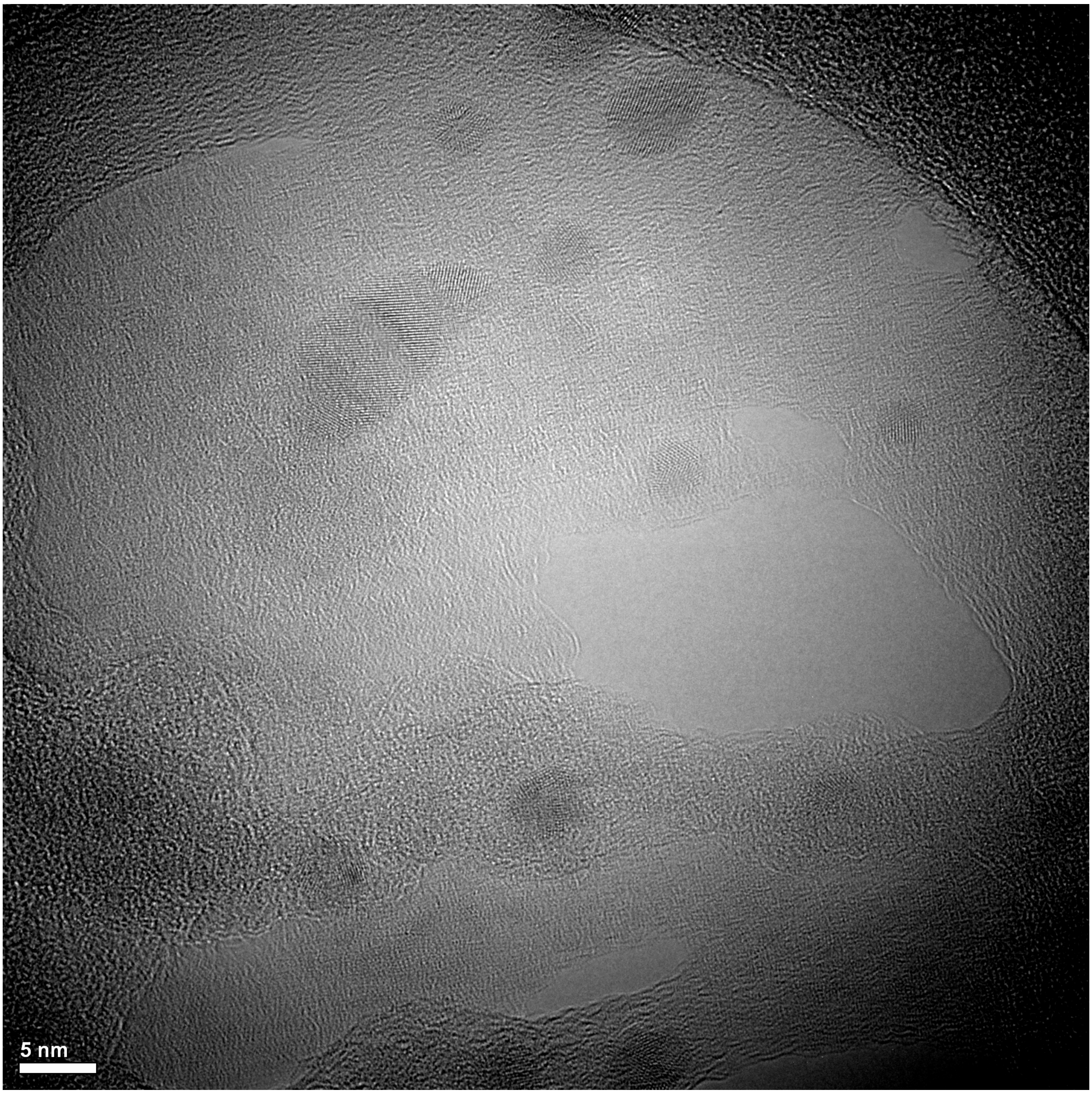

Figure S3: Cluster of nanocrystals at the periphery of the ion track at the irradiation dose of 5×10^{11} ion/cm^2, ion energy is 167 MeV.
